# Molecular-Dynamics Study on the Impact Energy Release Characteristics of Fe–Al Energetic Jets

**DOI:** 10.3390/ma14185249

**Published:** 2021-09-13

**Authors:** Qiang Li, Chunlan Jiang, Ye Du

**Affiliations:** 1 College of Mechatronic Engineering, North University of China, Taiyuan 030051, China; liqiang1170@126.com (Q.L.); dy_ibc@nuc.edu.cn (Y.D.); 2 State Key Laboratory of Explosion Science and Technology, Beijing Institute of Technology, Beijing 100081, China

**Keywords:** energetic jets, impact loading, energy release, molecular dynamics, multi-scale

## Abstract

Fe–Al energetic material releases a large amount of energy under impact loading; therefore, it can replace traditional materials and be used in new weapons. This paper introduces the macroscopic experiment and microscopic molecular-dynamics simulation research on the energy release characteristics of Fe–Al energetic jets under impact loading. A macroscopic dynamic energy acquisition test system was established to quantitatively obtain the composition of Fe–Al energetic jet reaction products. A momentum mirror impacting the Fe–Al particle molecular model was established and the microstructure evolution and impact thermodynamic response of Fe–Al particles under impact loading were analyzed. The mechanism of multi-scale shock-induced chemical reaction of Fe–Al energetic jets is discussed. The results show that the difference in velocity between Fe and Al atoms at the shock wave fronts is the cause of the shock-induced reaction; when the impact strength is low, the Al particles are disordered and amorphous, while the Fe particles remain in their original state and only the oxidation reaction of Al and a small amount intermetallic compound reaction occur. With the increase of impact strength, Al particles and Fe particles are completely disordered and amorphized in a high-temperature and high-pressure environment, fully mixed and penetrated. The temperature of the system rises rapidly, due to a violent thermite reaction, and the energy released by the jet shows an increasing trend; there is an impact intensity threshold, so that the jet release energy reaches the upper limit.

## 1. Introduction

The large amounts of energy released by energetic materials enables the replacement of conventional materials in new types of weapons [1,2,3]. Among them, the rapid reaction phenomenon and high exothermicity between the shock-induced chemical reaction (SICR) of the Fe–Al composite energetic material attract an increasing number of researchers [4,5,6,7]. The material is insensitive to friction, combustion and explosion under normal conditions. However, under a strong impact load, the impact energy drives the Fe phase and the active Al phase to react violently, releasing a large amount of energy. Therefore, applying this energetic material to the liner can form an energetic jet and greatly increase the damage power [8].

Recently, substantial progress had been made in the field of preparation and impact deformation of Fe–Al energetic composite materials. Du et al. [9] coated nano Al powder based on the chemical liquid deposition method and chemical vapor deposition method and prepared a new type of metal Fe/Alp composite powder, which improved the stability and energy release efficiency of nano Al powder. Airiskallio et al. [10] experimentally determined that Fe–Al intermetallic compounds have outstanding antioxidant properties. Wang et al. [11] prepared Fe–Al micro/nanocomposite particles with core-shell structures and found that the thermal reactivity of the Fe–Al micro/nanocomposite powder was significantly higher than that of the raw Al powder by differential scanning calorimetry (DSC) analysis. Wang et al. [12] studied the influence of composition ratio on the reaction heat of Fe–Al energetic materials through DSC scanning and SEM microstructure and optimized the Fe–Al composition scheme based on the highest energy release efficiency. Andryushchehko et al. [13] applied a pulsed laser to the surface of an Fe–Al–C alloy containing diamond crystals to form spherical graphite particles with a size of 100–300 μm and obtained the law of the effect of pulsed laser on the surface of Fe–Al–C alloy samples containing diamond crystals. Zamanzade et al. [14] used nanoindentation technology to evaluate the contribution of single vacancy, double vacancy and triple defects of FeAl intermetallics to investigate the transformation of mechanical properties. The study found that, in samples with double vacancies, compared with single vacancies, the increase in hardness was more obvious.

Understanding the mechanism of the energy released by the Fe–Al energetic jet under impact loading is a key link to promote its application. However, limited by testing conditions, observing the macroscopic experimental phenomena is obviously not enough to reveal the mechanism of the material at the micro level. The impact compression experiment has significant limitations in detecting the deformation in the particle mixture and the microscopic details of the large-scale mixing flow. In order to make up for the deficiency of experimental observation methods, scholars try to use molecular-dynamics simulation methods to study the physical phase transition process of Fe-based materials after impact loading. Guo et al. [15] used molecular-dynamics methods to study the structural phase transition of Fe–Al materials under high-rate compression loading conditions and analyzed the changes in pressure and temperature with strain during the material loading process. Wang et al. [16] studied the plasticity and phase transition of Fe under high pressure based on the embedded atom model. For the first time, the impact plastic behavior of Fe was observed on the atomic scale. Based on the first principles, Lu et al. [17] conducted a non-equilibrium molecular-dynamics simulation of polycrystalline Fe and obtained the phase transition mechanism of Fe under impact compression. Huang et al. [18] used the non-equilibrium molecular-dynamics method to simulate the shock compression response of a single crystal Fe model with multiple dislocation structures. It is found that under high-intensity impact, plastic deformation in local areas is beneficial to the occurrence of phase transformation. Gunkelmann et al. [19] used the improved potential function of Fe to study the plastic deformation and phase transformation of Fe under impact loading. It was found that plastic changes such as dislocations occurred. Yang et al. [20] analyzed the dependence of the incident energy of deposited atoms on the growth configuration of Fe–Al nanoparticles. The impact deposition of Al (or Fe) atoms on the rhombohedron of Fe (or the truncated octahedron of Al) nanoparticles was investigated by performing a molecular-dynamics simulation using the embedded atom method.

The current research on the SICR of Fe–Al energetic materials is mainly focused on observing the physical microstructure of the static initial and final states of the powder mixture, but the mechanism of the chemical reaction process of the energy release under high strain rate loading is still not clear. This study aims to fill the gap in research on the energy release mechanism of Fe–Al energetic materials when applied to jets.

In this paper, by designing energy-harvesting experiments, the impact energy-release characteristics of Fe–Al energetic jets under different impact intensities are discussed. Molecular-dynamics simulation methods are used to analyze the impact compression response process and SICR mechanism of Fe–Al composites. The research reveals the impact energy-release mechanism of Fe–Al energetic jets from the macro–micro multi-scale.

## 2. Methods of Simulation

### 2.1. Experimental Design and Scheme

**Figure 1:**
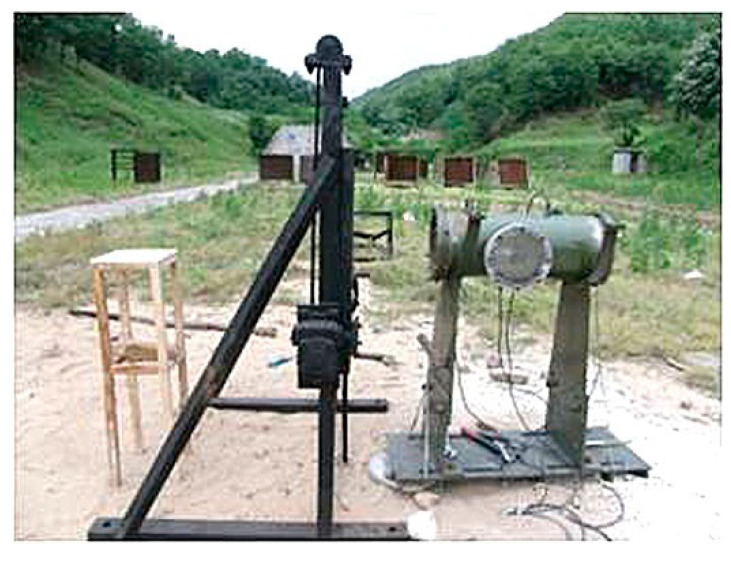
Field experimental device.

**Figure 2:**
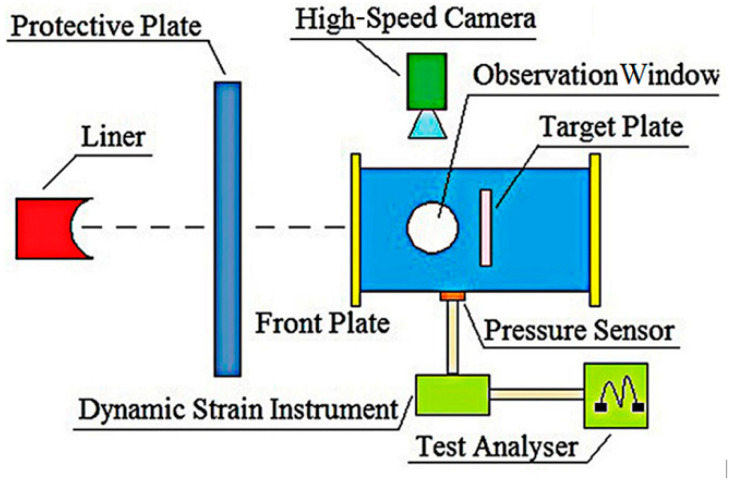
Schematic diagram of the energy acquisition system.

The Fe–Al energetic jet-dynamic energy-acquisition test system was designed and the impact energy release characteristics of the Fe–Al energetic jet were studied by experimental means. By adjusting the thickness of the target plate, the jet was loaded with different impact energy. The XRD analysis method was used to observe and test the composition of the residual substance in the container. The field experimental device is shown in Figure 1 and the experimental system is shown in Figure 2. The specific experimental devices and methods of data processing have been elaborated in my previous articles [21] and are not repeated here.

### 2.2. Molecular-Dynamics Simulation Method

**Figure 3:**
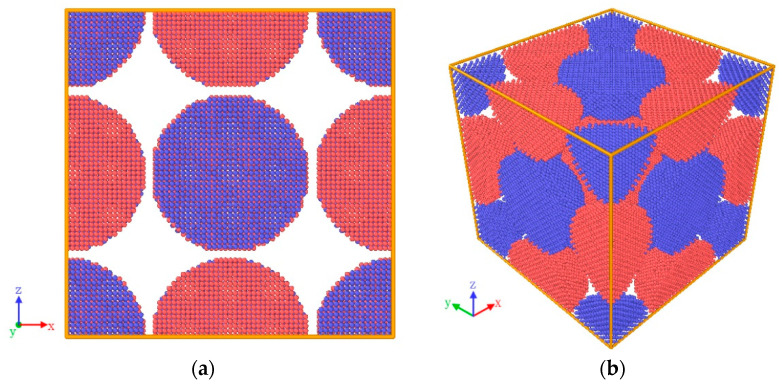
The model of the initial unit (Fe atoms are colored in red and Al atoms are colored in blue) (**a**) Front view (**b**) Oblique view.

To further reveal the mechanism of the macroscopic impact energy-release phenomenon of Fe–Al energetic jets, the molecular-dynamics simulation was carried out. The embedded atom method (EAM) proposed by Mendelev et al. and the potential function between Al and Fe proposed by Eunkoo et al. [22] are used to describe the interaction between Fe–Al metal atoms. The LAMMPS molecular-dynamics software is used to simulate the relaxation and shock response process of Fe–Al particles. In order to facilitate the homogeneously mixing of Fe–Al particles, spherical Fe particles and spherical Al particles are arranged and stacked according to the cubic crystal structure. In order to facilitate the alternating and uniform mixing of Fe–Al particles, the initial unit is established by arranging and stacking spherical Fe particles and spherical Al particles according to the cubic crystal structure [23]. Each unit contains 4 Fe particles and 4 Al particles. The lattice points in the two kinds of particles create single Fe atoms and Al atoms, respectively, as shown in Figure 3. The initial diameters of Fe particles and Al particles are both 6 nm, the distance between adjacent particles is 0.25 nm and the unit size is 12.5 × 12.5 × 12.5 nm. It is expanded 1 time along the *x*-axis direction, 1 time along the *y*-axis direction and 10 times along the *z*-axis direction, constructing a particle mixing system containing 1.04 million atoms.

**Figure 4:**
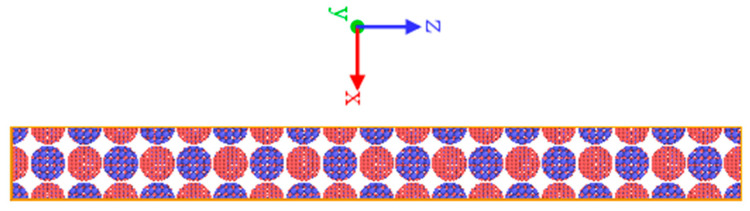
“Momentum mirror” impact model.

According to previous studies, the energetic-type cover produces an impact temperature rise and a plastic temperature rise during the process of forming an energetic jet after undergoing a process of crushing and stretching and the final temperature can reach 700 K [24]. In order to simulate the state of the energetic jet accurately before it hits the target plate, the initial case is relaxed at 700 K. First, the model is relaxed under the conjugate gradient method to minimize the energy, then the temperature is maintained at 700 K under the isothermal–isobaric ensemble (NPT) and, finally, under the microcanonical ensemble (NVE), for 300 ps. The relaxed model is used as the initial state of impact simulation. The method of “momentum mirror” is used to create an infinite mass piston, which moves to the Z axis at a speed of Up and applies impact loading on the Fe–Al mixed particle model. As shown in Figure 4, the loading speed range is 400–4000 m/s. The particles obtain different impact energy by changing the impact velocity and the simulation results obtained are compared with the macroscopic experiment of the corresponding impact energy. The entire impact process is carried out in the NVE with a time step of 100 ps. A free boundary is applied on the Z axis and periodic boundary conditions are applied in the other directions. The software OVITO (Version 3.4.4) is used to visualize and analyze the simulation results.

## 3. Results and Discussion

### 3.1. Impact Reaction Product Composition

**Figure 5:**
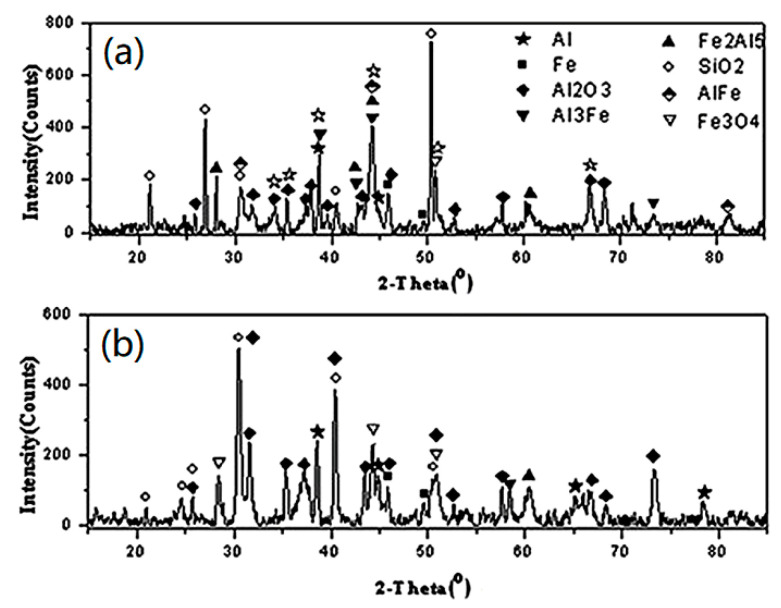
XRD patterns of the reaction products with different thickness targets: (**a**) 2-mm target; (**b**) 5-mm target. This picture is taken from “Li, Q.; Du, Y. Experimental Study on the Energy-Release Characteristics of Fine-Grained Fe/Al Energetic Jets under Impact Loading. *Materials* **2019**, *12*, 3317” [21].

Figure 5 shows the XRD patterns of the recovered products. The XRD detection method refers to the paper by Gorji N.E. [25].

It can be seen, from Figure 5, that, in the low-impact strength-recovered products, the elemental Al content is the highest. Additionally, small amounts of intermetallic composites are found and no substantial amount of Fe_3_O_4_ is formed, indicating that the Al oxidation and Fe–Al intercalation reactions occur mainly under low-impact strength conditions. A high-impact strength results in the recovery of only a small amount of metallic elements and composites, most of which are Al_2_O_3_ and Fe_3_O_4_, indicating a great quantity of oxidative reactions and thermite reaction of energetic substances in Fe–Al jet is induced by high-impact strength, as shown in Equations (1)–(3). The results shown in Figure 5 explain the phenomenon of more energy released by a Fe–Al energetic jet under high-impact strength from mesoscopic scale.

Al + 3/4O_2_ = 1/2 Al_2_O_3_ (1)

Fe + 2/3O_2_ = 1/3 Fe_3_O_4_ (2)

Al + 3/8Fe_3_O_4_ = 9/8Fe + 1/2Al_2_O_3_ (3)

### 3.2. Evolution of Particle Microstructure

**Figure 6:**
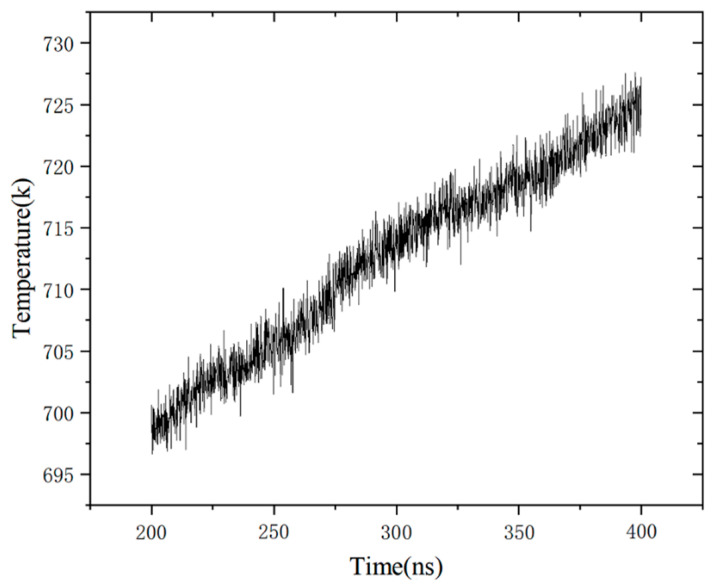
Regular pattern of temperature changed after relaxation.

The regular pattern of temperature change obtained by the unit model with 700 K temperature relaxation is shown in Figure 6. The temperature increased slightly by 25 K within 200 ps. It shows that the Fe–Al energetic material is in a metastable state after forming a jet and the spontaneous reaction occurs slowly.

**Figure 7:**
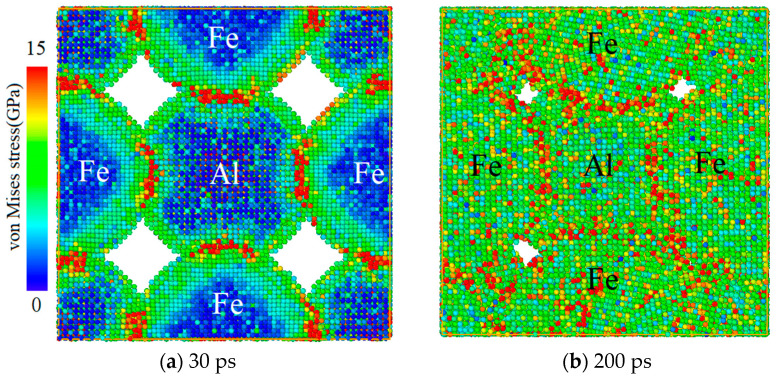
Stress distribution during model relaxation.

The atoms are colored with local von Mises stress and the stress distribution during the relaxation process is shown in Figure 7. It is found that when the relaxation lasts for 30 ps, as the temperature increases, the kinetic energy of the particles increases and the particles of the two elements squeeze and collide at the interface, with a stress value of 15 GPa. In contrast, little change occurs for the stress in the center of particle. When the relaxation lasts for 200 ps, the boundary between the particles of the two elements is severely deformed and the gap between the particles is significantly reduced or it even disappears. The atoms in the inner regions of the particles also begin to squeeze each other, forming a GPa-level stress, and the interpenetration of atoms of different elements appears at the boundary. It is speculated that, during the jet-formation stage, an intermetallic compound reaction occurs between Fe and Al atoms, but most of the material activity is still maintained. Due to the low-yield strength of Al particles, they can no longer maintain a spherical shape under extrusion and collision, so the internal stress of the particles is not uniformly distributed.

**Figure 8:**
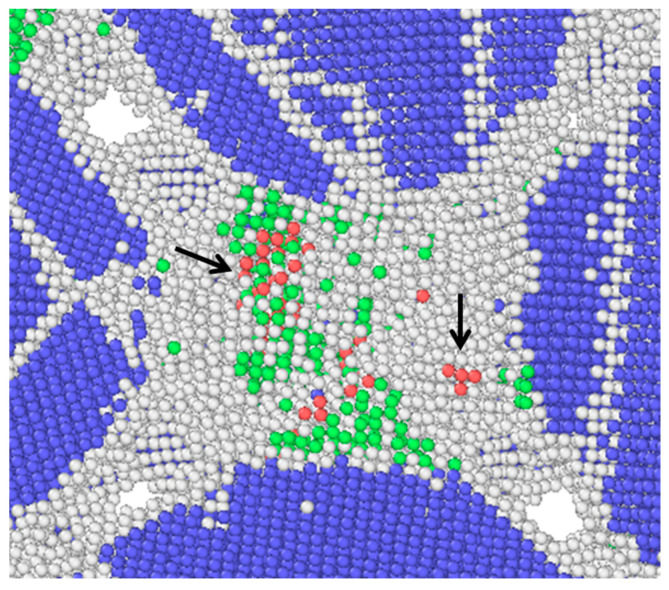
Microstructure after relaxation (FCC, BCC, HCP and unclassified atoms are colored in green, bule, red and white, respectively).

The microstructure of the unit after relaxation is shown in Figure 8. Obvious strip defects can be found inside the Fe particles, but no metastable phase of FCC. A small amount of HCP phase transition can be found inside the Al particles, forming several asymmetric slip surfaces, as indicated by the arrows in Figure 8. The relaxed configuration is similar to the state of the porous material after impact compression. It shows that, even if there is no external force, when the high-temperature jet is formed, the Al and Fe particles form uneven local stress during the extrusion process, resulting in a large number of lattice defects.

**Figure 9:**
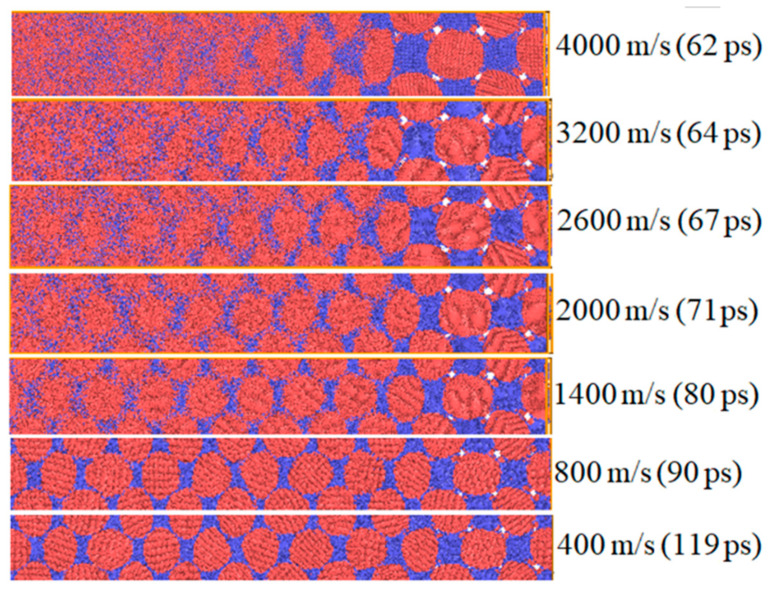
Particle morphology under different impact strength.

Figure 9 shows the morphology of Fe–Al particles under different impact strengths. When the impact velocity is less than 800 m/s, as the shock wave propagates in the model, the Al particles are severely deformed and fill the pores with the increasing density of the overall particle. This is because, at a low-impact velocity, the relaxation temperature (725 K) plus the impact temperature rise exceeds the melting point of Al, forcing the Al particles in a molten state. The pre-existing gap defects between particles accelerate the flow and deformation of Al particles. The melting point of iron particles is higher than that of Al, so they do not deform much at low-impact speeds. In contrast, little change occurs in the morphology of Fe particles. When the impact velocity is greater than 1200 m/s, the shape of Fe and Al particles changes from a sphere to an ellipsoid at the front of the shock wave. The greater the impact velocity, the more serious the deformation. As the impact velocity increases behind the wave front, the Al particles and Fe particles become increasingly disordered with drastic plastic deformation, permeating and fusing with each other.

**Figure 10:**
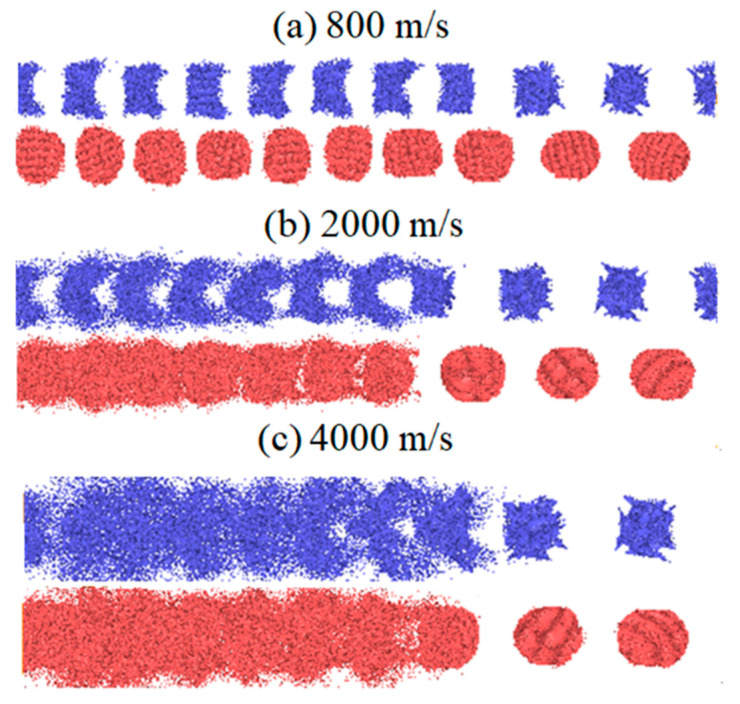
Deformation states of two particles under different impact speeds (Fe particles are colored in red and Al particles are colored in blue).

In order to compare the deformation behaviors of particles during impact compression, the deformation states of the two particles are extracted separately along the Z axis, as shown in Figure 10. When the impact velocity is 800 m/s, the deformation extent of Al particles is greater than that of Fe particles. Al particles show a morphology compressed by shock waves, but still remain an ordered arrangement. The morphology of Fe particles is changed from sphere to ellipsoid. When the impact velocity is 2000 m/s, the Al particles are driven by high stress to form obvious high-speed jets on both sides, forming local area deformation. The ejected Al atoms penetrate through the Fe particles and reach the adjacent Al particles, which induces the activation of the Al/Fe interface, which is more conducive to the occurrence of chemical reactions. The Fe particles do not form an obvious jet, but there is still a considerable amount of Fe atoms peeled off the surface of the particles and fused with the adjacent Al particles, showing that the morphology of Fe particles is obviously loose. When the impact velocity is 4000 m/s, both Al and Fe are severely disordered and amorphized and the original morphology cannot be identified. The moving distance of the atoms increases significantly and the two kinds of atoms are deeply fused, which are the conditions to induce chemical reactions.

**Figure 11:**
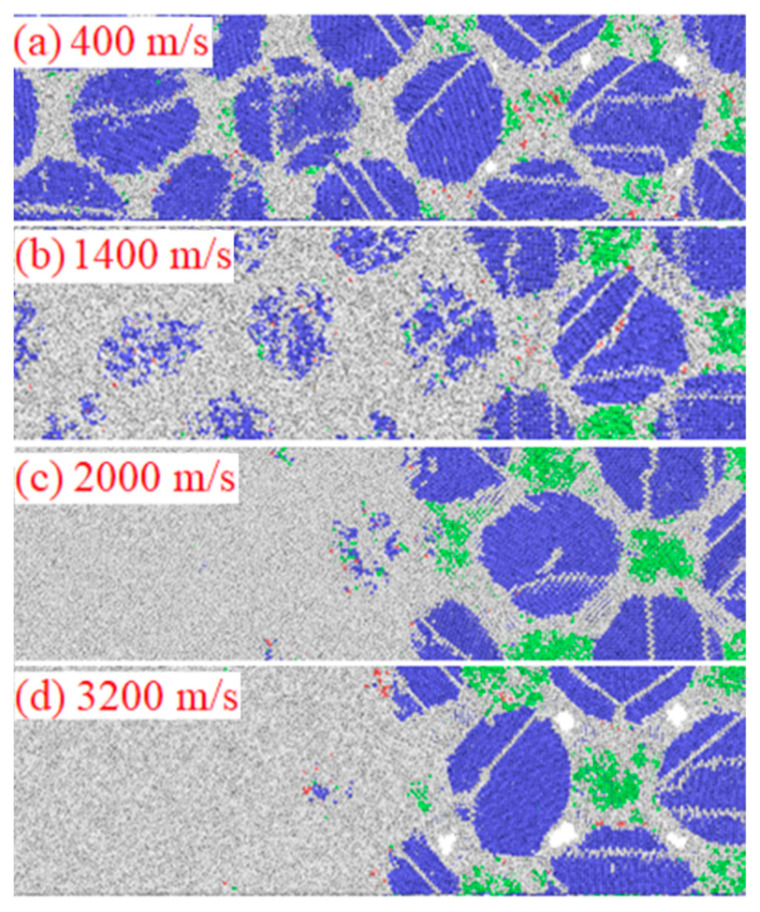
Microstructure of wave fronts at different impact velocities.

Figure 11 shows the microstructure of wave fronts at different impact velocities. It can be found that, when the impact velocity is less than 400 m/s, except for a small part of the Al particles in the impacted area which maintain the FCC structure, the rest become amorphous. The slip planes that appeared during relaxation basically disappear and the transition from slip to twinning does not occur. A certain number of disordered atoms appears inside Fe particles, but most of them maintain the BCC structure. This is because the particle temperature is lower than 1200 K, as shown in Figure 18 (mentioned below), which does not meet the conditions for forming the γ-Fe FCC structure. As the impact velocity increases to 1400 m/s, the Al particles are further disordered and the FCC structure basically disappears. A large number of defects appears in the Fe particles and only about half of the BCC structure remains. When the impact velocity is greater than 2000 m/s, the amorphous transformation of Fe is completed in the narrow area of the shock-wave front and the impact area is basically completely amorphous. Comparing the particle morphology under different impact strengths (as shown in Figure 9), it can be found that, when the impact velocity is greater than 1400 m/s, first, the Al particles melt because the temperature exceeds the melting point and the liquid Al quickly fills the defect spaces between the particles. Next, part of the Fe particles transforms into an amorphous state (the number of transformations is proportional to the impact speed), which is further refined and mixed with liquid Al atoms. The mixing process is different from the atomic second-level slow diffusion under static conditions, but, when the shock wave front is loaded, the two elements are mixed at an ultra-fast ps level. It shows that the amorphization of Fe particles under impact loading can greatly increase the reaction rate, which verifies the rationality of the hypothesis of high diffusion rate in SICR [26].

### 3.3. Impact Response

**Figure 12:**
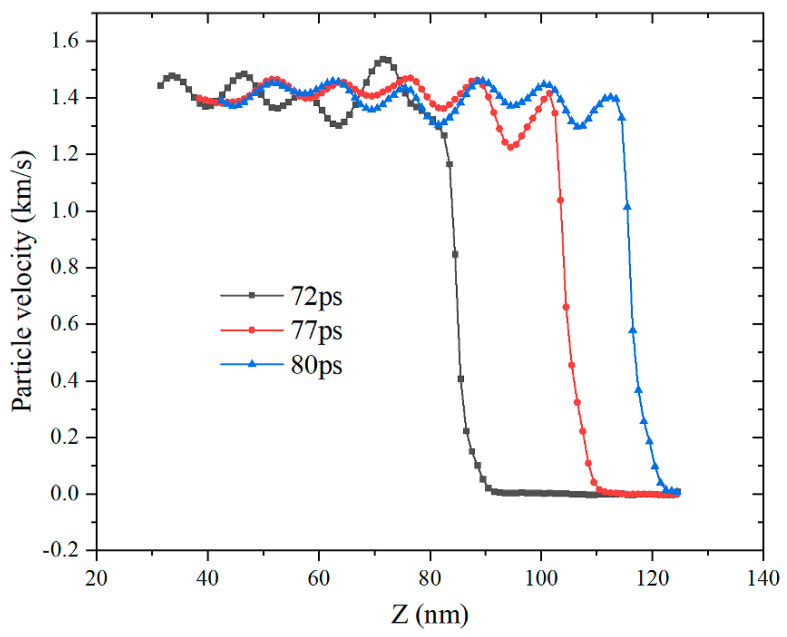
Distribution of particle velocity at different moments of impact velocity (1400 m/s).

**Figure 13:**
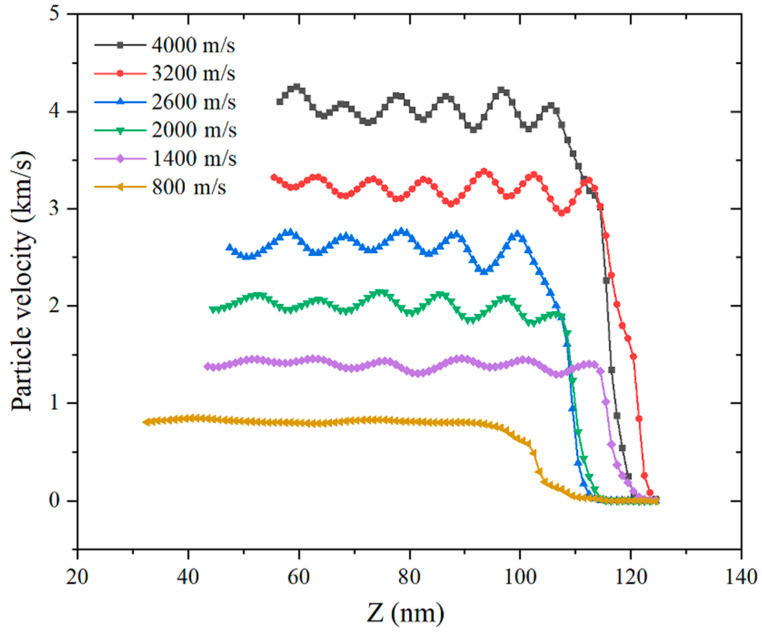
Comparison of particle velocities with different impact velocities.

Figure 12 shows the particle velocity waveforms at different moments when the impact velocity is 1400 m/s. Since there are differences in the types and speed of motion of atoms in the same sub-regions, this figure refers to the average speed in the sub-regions. It can be seen that the particle velocity in the impact loading area presents periodic oscillations. Under the same impact velocity, the particle velocity of the shock-wave front passing through different areas has basically the same changing rule. The particle velocity increases rapidly where the wave front reaches and the particle velocity at a certain distance behind the wavefront is higher than when the front passes by. It shows that the Al–Fe mixed particles induce a reaction after being loaded by shock, release energy and accelerate the movement of the particles. As the impact loading speed increases, the particle velocity shows an increasing trend, as shown in Figure 13.

**Figure 14:**
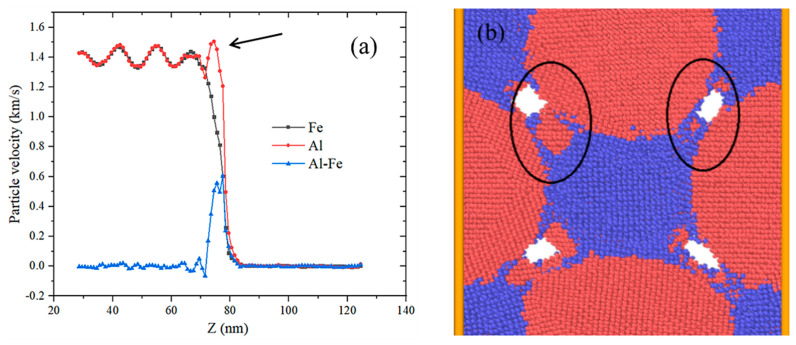
Velocity difference of Al and Fe particles: (**a**) comparison of the velocity of Al and Fe particles; (**b**) an enlarged view of the particle morphology at the arrow shown in (**a**).

Since the particle velocity dispersion (PVD) is a possible mechanism to explain the SICR, it is necessary to extract the particle velocities of Al particles and Fe particles separately for comparison. As shown in Figure 14, at a certain distance behind the wave front, the particle velocities of the two elements are basically the same, but at the front of the shock wave, there is a difference in velocity. The particle velocity of Al particles is much higher than that of Fe particles. As shown in the black circle in Figure 14b, under high-impact strength, Al atoms are ejected from the particles to form high-speed moving particles, passing through the Fe particles, so that part of the Fe atoms are peeled off the surface, the particles are refined and a new contact area is formed at the same time, which realizes recombination and admixture of two kinds of atoms and creates the conditions of SICR.

**Figure 15:**
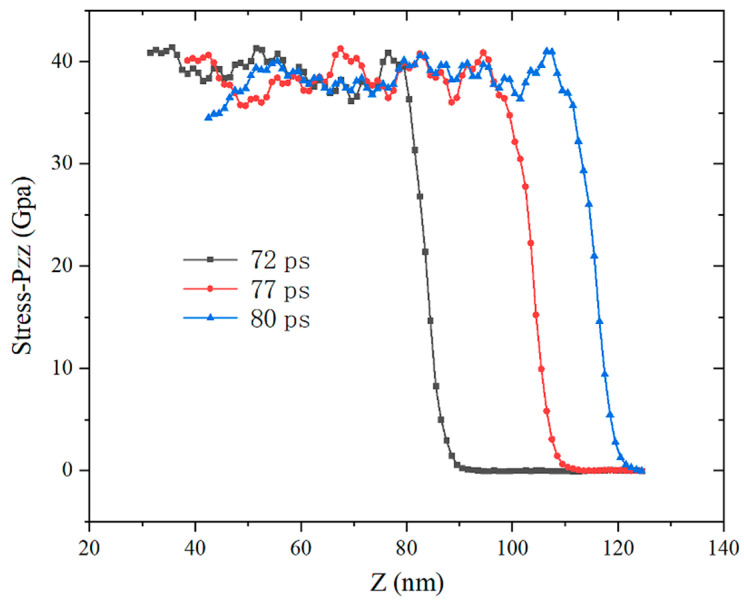
Impact velocity 1400 m/s stress waveform at different moments.

**Figure 16:**
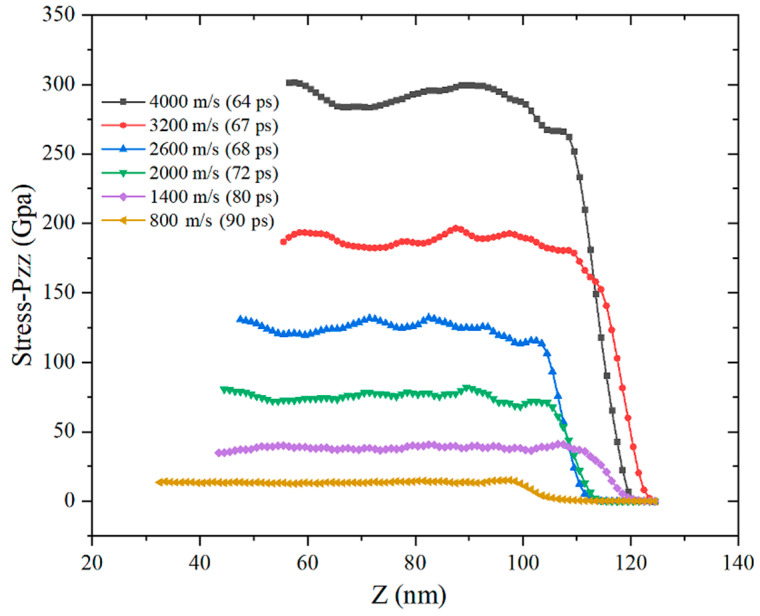
Stress waveforms at different impact speeds.

The particles in the impact compression area collide with each other to form the average stress in the *Z*-axis direction, as shown in Figure 15 and Figure 16. Compared with the particle velocity waveform diagram, the periodic oscillation of the stress waveform is weakened due to the randomness of the collision. Similar to the particle velocity waveform, as the impact velocity increases, the Z-axis stress shows a nonlinear increasing trend. The greater the impact velocity, the greater the stress increase. When the impact velocity is 4000 m/s, the instantaneous particle stress can reach 300 GPa, which has far exceeded the yield limit of the two elements.

### 3.4. Thermal Effect

**Figure 17:**
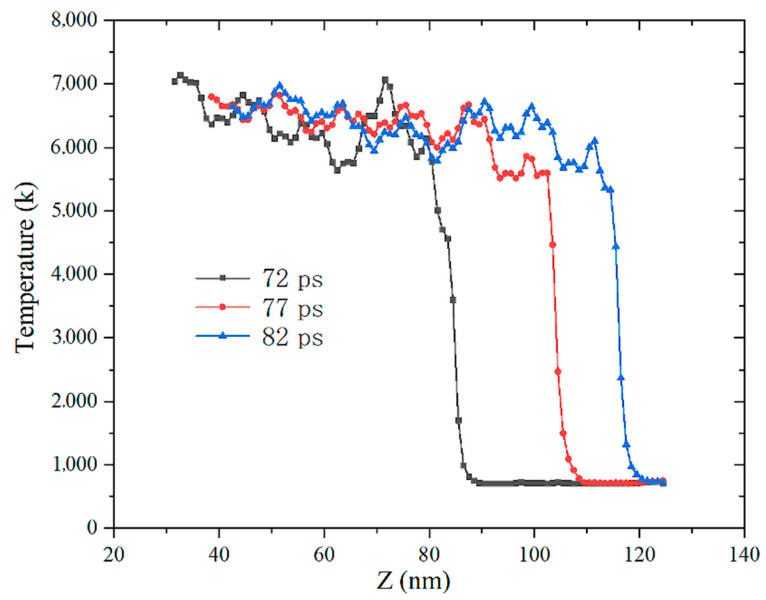
Temperature distribution at 1400m/s impact velocity.

**Figure 18:**
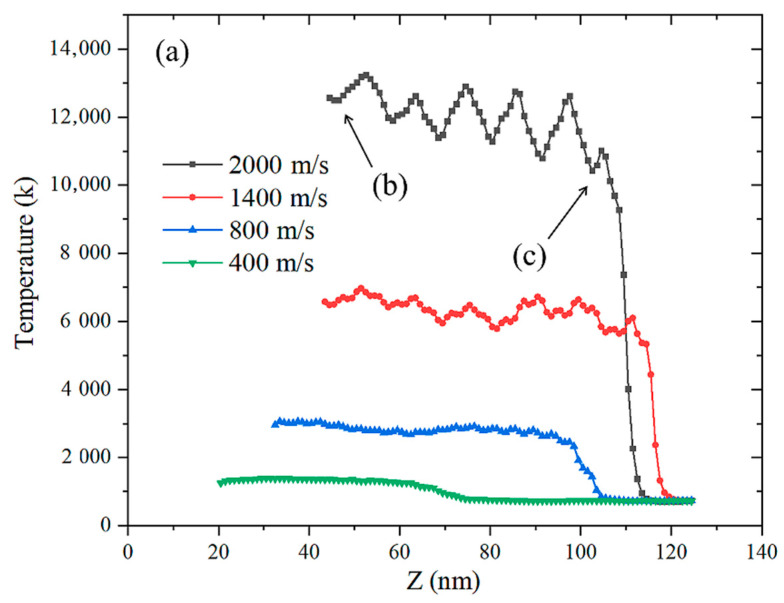

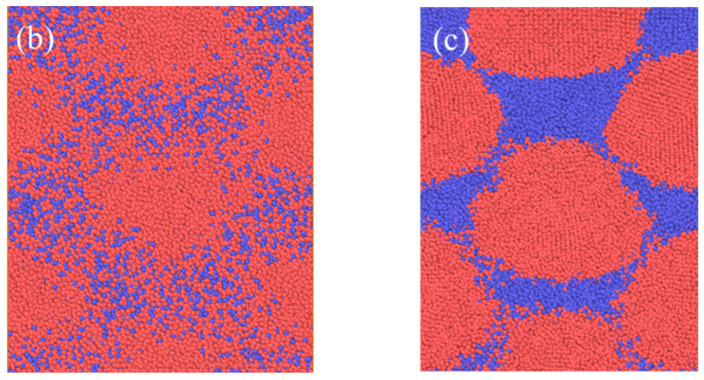
Temperature distribution at different impact speeds: (**a**) is the temperature distribution of the particles; (**b**,**c**) is the enlarged particle morphology of the arrows in (**a**).

When the material is subjected to impact loading, the temperature rise caused by the impact, the plastic deformation and the chemical reaction occur and the three cause the temperature rise of the system. It can be seen from Figure 17 and Figure 18 that, when the impact velocity is 400 and 800 m/s, as the shock wave propagates in the *Z*-axis direction, the temperature distribution remains straight, basically unchanged. It shows that, under the condition of impact strength below 800 m/s, the particles in the impacted area repeat the same plastic deformation temperature rise only due to impact compression and no chemical reaction is induced. When the impact velocity is 1400 and 2000 m/s, the temperature distribution fluctuates violently, which indicates that the temperature of the system rises rapidly due to the exothermic reaction of the impact model, showing obvious SICR characteristics.

### 3.5. Analysis of Impact Energy-Release Mechanism

**Figure 19:**
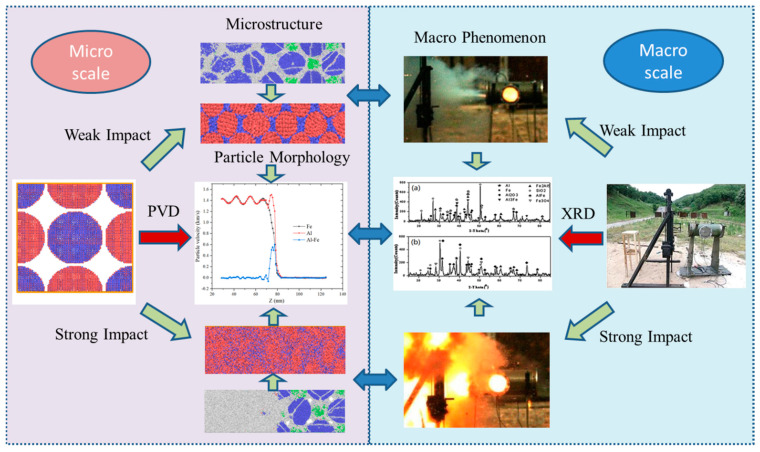
Mechanism of Fe–Al energetic jet induced reaction.

Comparing the phenomenon of the Fe–Al energetic jet macroscopic impact energy-release experiment and the microscopic molecular-dynamics simulation results, it can be found that, as the impact strength increases, the impact induced reactivity of the Fe–Al energetic jet increases, but there is an impact strength threshold, which makes the energy released by the energetic jet reach the upper limit. According to the XRD analysis of the recovered product, it is speculated that the oxidation reaction of Al and the compound reaction between Al and Fe mainly occur under low-impact strength and the oxidation reaction of Fe and thermite reaction occur under high-impact strength. Through micro-molecular-dynamics simulation, it is found that only Al particles are disordered and amorphized under low-impact strength. The ejecta of Al fills the gaps between the particles and increases the contact area. Therefore, Al takes the lead in the oxidation reaction. The Fe particles maintain the original BCC structure and are surrounded by molten Al particles, resulting in a small amount of intermetallic reaction. When the Fe–Al energetic jet is loaded with high-impact strength, Al particles and Fe particles are completely disordered and amorphized in a high temperature and high-pressure environment and fully mixed and penetrated, which greatly improves the reaction rate. At the same time, the Fe particles are further refined (the surface oxide layer may be broken), exposing a new surface and being oxidized again. Due to the full contact with Al atoms, the temperature exceeds 1500 K, which induces a violent thermite reaction. Through the above analysis, it can be found that the conclusions obtained by the microscopic simulation are completely consistent with the results of the macroscopic phenomenon and meso-component analysis, revealing the mechanism of the Fe–Al energetic jet impact-induced reaction from a multi-scale perspective, as shown in Figure 19.

## 4. Conclusions

In this paper, macroscopic experiments and microscopic molecular-dynamics simulation studies on the energy-release characteristics of Fe–Al energetic jets under impact loading were carried out. A macroscopic dynamic energy acquisition test system was established to quantitatively acquire the overpressure formed by the energy released by the Fe–Al energetic jet. A molecular impact model of Fe–Al particles was established and the microstructure evolution and impact thermodynamic response of Fe–Al particles under impact loading were analysed. Comparing with the results of macroscopic experiments, microstructure analysis and microscopic simulation, the mechanism of the Fe–Al energetic jet multi-scale impact-induced reaction was discussed. The following specific conclusions can be drawn:

(1) The reactivity of the Fe–Al energetic jet is related to the impact strength. As the impact strength increases, the energy release of the jet increases. There is an impact strength threshold, which makes the energy released by the jet reach the upper limit, and the maximum response rate is 95.3%.
(2) When the high-temperature jet forms and does not touch the target plate, the Al and Fe particles are squeezed and deformed and atom penetration of different elements appears at the boundary, forming uneven local stress at the GPa level and a large number of lattice defects are generated at the same time.
(3) The speed difference between Fe and Al atoms is formed at the shock wave front. High-speed Al atoms are ejected from the particles and pass through the Fe particles, causing part of the Fe atoms to peel off from the surface. The particles are refined and a new contact area is formed at the same time. The alternate recombination of the two kinds of atom creates conditions for impact-induced reactions.
(4) When the impact strength is low, the temperature distribution along the Z-axis of the model remains a straight line, indicating that the particles in the impact area only repeat the same plastic deformation temperature rise due to impact compression. When the impact strength is high, the temperature distribution fluctuates violently, which indicates that the temperature of the system rises rapidly due to the exothermic reaction of the impact model, showing obvious SICR characteristics.
(5) The mechanism of the Fe–Al energetic jet impact-induced reaction is as follows: Under low-impact strength, only Al particles become disordered and amorphous, which increases the contact area, and Al undergoes an oxidation reaction. By comparison, the Fe particles maintain the original BCC structure and are surrounded by molten Al particles, resulting in a small amount of intermetallic chemical reaction. With high-impact strength, Al particles and Fe particles are completely disordered and amorphized in a high temperature and high-pressure environment and fully mixed and penetrated. The temperature of the particles exceeds 1500K, which induces a violent thermite reaction.

## Data Availability

Due to privacy or moral restrictions, the data is not publicly available.
